# Trace Aflatoxins Extraction in Pistachio, Maize and Rice Based on β-Cyclodextrin-Doped Cu-Carboxylated Graphene Oxide Nanocomposite

**DOI:** 10.3390/toxins17110562

**Published:** 2025-11-17

**Authors:** Amr A. Yakout, Wael H. Alshitari, Hassan M. Albishri, Faten M. Ali Zainy, Adel M. Alshutairi

**Affiliations:** 1 Department of Chemistry, College of Science, University of Jeddah, Jeddah 23218, Saudi Arabia; 2 Chemistry Department, Faculty of Science, Alexandria University, Alexandria 21526, Egypt; 3 Department of Chemistry, Faculty of Science, King Abdulaziz University, P.O. Box 80203, Jeddah 21589, Saudi Arabia

**Keywords:** aflatoxins, rice, maize, pistachio, carboxylated graphene oxide, β-cyclodextrin, high performance liquid chromatography (HPLC)

## Abstract

Aflatoxins remain among the most challenging food contaminants to monitor due to their structural diversity, low abundance, and the chemical complexity of cereal- and nut-based matrices. In this study, a multifunctional Cu/β-cyclodextrin@carboxylated graphene oxide (Cu/β-CD@CGO) nanocomposite was synthesized through a green, two-step procedure and employed as a high-affinity nanosorbent for trace extraction of AFB_1_, AFB_2_, AFG_1_, and AFG_2_. The architecture integrates three complementary components: β-cyclodextrin for inclusion-driven molecular recognition, copper nanoparticles that establish coordination interactions with lactone-bearing aflatoxins, and CGO nanosheets that supply extensive π-rich surfaces and abundant carboxyl functionalities. Comprehensive characterization (FTIR, Raman, XPS, SEM, EDX-mapping, and HRTEM) confirmed the formation of a uniform, porous hybrid network. Under optimized d-SPE conditions, the nanocomposite enabled quantitative recovery (92.0–108.5%) of aflatoxins from pistachio, maize, and rice extracts while achieving sub-ng kg^−1^ detection limits and excellent reproducibility. The results demonstrate that the Cu/β-CD@CGO platform provides a robust, selective, and sustainable alternative to conventional immunoaffinity or polymeric sorbents, offering strong potential for routine surveillance of aflatoxins in complex food systems.

## 1. Introduction

Consumers and producers worldwide have developed a greater understanding of and interest in food quality and supply owing to many food products that include different types of fungi. Mycotoxins are a family of dangerous fungal secondary metabolites such as *Fusarium*, *Aspergillus*, and *Penicillium* [1]. According to the Food and Agriculture Organization (FAO), the longstanding estimate of 25% of global food-crops being contaminated by mycotoxins is still frequently cited; however, more recent analyses suggest that the actual incidence of detectable mycotoxin contamination may be as high as 60–80% of staple crops under tropical or warm conditions, making these toxins among the most widespread contaminants in staple crops [2]. They have been identified as a group of carcinogens, teratogens, and mutagens to humans [3,4,5,6]. There are currently over 20 different types of aflatoxins (AFs) known to exist, and four of them, AFB1, AFB2, AFG1, and AFG2, are easily contaminated in the environment [7]. AFs are naturally occurring compounds that share pentacyclic skeletons with coumarin and dihydrofuran [8]. According to studies, coumarin is closely linked to carcinogenicity, and the terminal double bond on the dihydrofuran ring is more hazardous [9]. More than 100 nations have set a maximum AFB1 restriction of less than 20.0 μg kg^−1^ in various foods, and the World Health Organization and the International Agency for Research on Cancer both classified AFB1 as a Class I carcinogen [10]. The European Commission has set a maximum concentration of AFB1 in rice and edible oil of 2.0 μg kg^−1^ and a maximum concentration of AFG1, AFG2, AFB1, and AFB2 of 4.0 μg kg^−1^ [11,12]. The 2020 edition of Tare Pharmacopoeia specifies that the permissible maximum of AFB1 in 24 medicinal goods is 5.00 μg kg^−1^, while the cumulative concentration limit for AFG1, AFG2, AFB1, and AFB2 is 10.0 μg kg^−1^ [12]. Numerous food and medical products, including seed materials, citrus peels, and kernel materials, have been reported to contain aflatoxins [13,14]. Consequently, regulating and eradicating AFs to ensure the quality and safety of medicinal and food homologous products has emerged as a significant global concern. Currently, it remains exceedingly difficult to directly and correctly identify trace AFs in a matrix of medicinal and culinary homologous items [15]. The challenges primarily stem from the substantial content variation and extensive polarity range of aflatoxins, as well as the numerous chemical types of interfering substances present in the intrinsic plant metabolome. As a result, the precise, sensitive, and effective measurement and recovery of trace AFs in different meals and feedstuffs is a popular subject. Numerous challenges arose in the direct detection of AFs in real samples at trace levels, primarily due to matrix interference and the limited sensitivity of available analytical methods [16]. The International Organization for Standardization (ISO), United States Pharmacopoeia, and Chinese Pharmacopoeia endorse the immunoaffinity column (IAC) approach for the enrichment and purification of AFs [17,18]. However, IAC presents drawbacks, including elevated costs, limited options for organic solvents, and vulnerable antibodies [17]. Therefore, it is essential to devise novel extraction and purification methodologies for AFs that are cost-effective, facilitate high analytical throughput, and exhibit no interference with antibodies and reagents [18].

Graphene oxide (GO), graphene (G), and carboxylated graphene oxide (CGO) are 2D carbon-based materials characterized by honeycomb and hexagonal architectures. These materials create an electron delocalization network through three sp^2^-hybridized links. The wonderful characteristics of these carbonaceous nanomaterials, including magnetic, electrical, and thermal properties, extensive surface areas, and exceptional mechanical strength, give them potential chances for various applications [19,20,21]. The ability of functionalized GO/G nanocomposites to prevent the re-stacking and aggregation of G/GO/CGO nanosheets has received considerable attention as potential nanosorbent materials for dispersed-solid phase extraction (d-SPE) [22,23,24,25,26,27,28,29,30,31].

Despite the growing number of graphene-based adsorbents reported for mycotoxin extraction, previous designs typically combined only one or two interaction mechanisms, such as β-CD inclusion or metal-assisted complexation. The present work introduces a new ternary architecture, Cu/β-CD@CGO, engineered to unify four synergistic adsorption pathways within a single nanostructure: Cu–lactone coordination, β-CD host–guest encapsulation, π–π stacking on CGO sheets, and extensive hydrogen bonding via carboxyl-rich surfaces. Unlike earlier Cu/β-CD/graphene derivatives, which relied on reduced graphene oxide and thus contained fewer oxygenated sites, the use of carboxylated graphene oxide in this study fundamentally alters the sorption chemistry by providing dense, accessible carboxyl groups that stabilize Cu-NPs, prevent nanosheet restacking, and enhance dispersive interactions with aflatoxins. This distinct structural configuration and its demonstrated analytical performance clearly differentiate the present nanocomposite from previously published systems.

**Figure 1:**
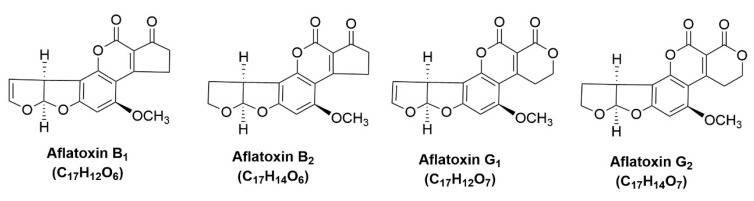
Chemical structures of target aflatoxins.

Cu-nanoparticle-doped β-CD functionalized CGO nanocomposite (Cu/β-CD@CGO) was therefore synthesized, integrating both cyclodextrin inclusion capabilities and CGO’s high surface activity. This synergistic platform was applied as a dispersive solid-phase extraction (d-SPE) sorbent prior to HPLC-DAD determination of four AFs (AFG1, AFG2, AFB1, and AFB2; Figure 1) in rice, maize, and pistachio samples. Comparative evaluations against commercial extraction columns demonstrated superior enrichment efficiency, underscoring the Cu/β-CD@CGO nanocomposite as a reliable and highly effective sorbent for AF monitoring in complex food matrices.

## 2. Results and Discussion

### 2.1. Surface Morphology and Characterization

**Figure 2:**
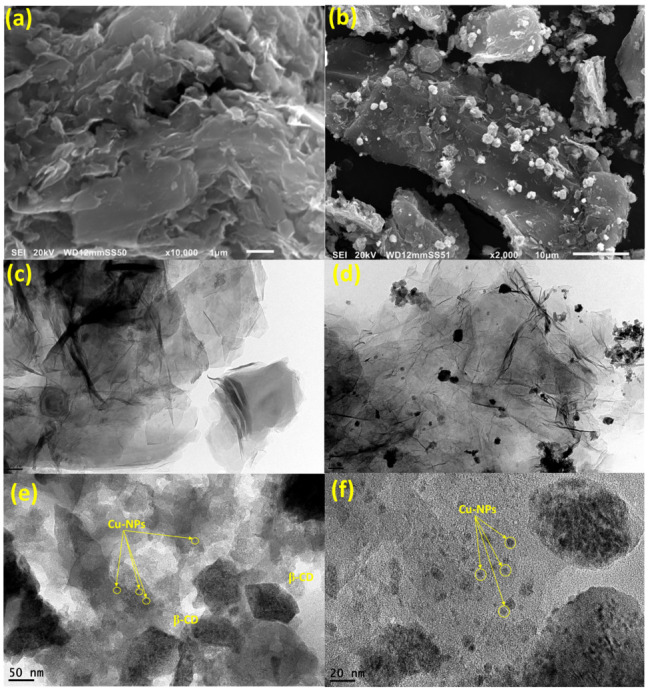
SEM (**a**,**b**) of CGO and Cu/β-CD@CGO, HRTEM of CGO (**c**) and Cu/β-CD@CGO (**d**–**f**) nanocomposites.

**Figure 3:**
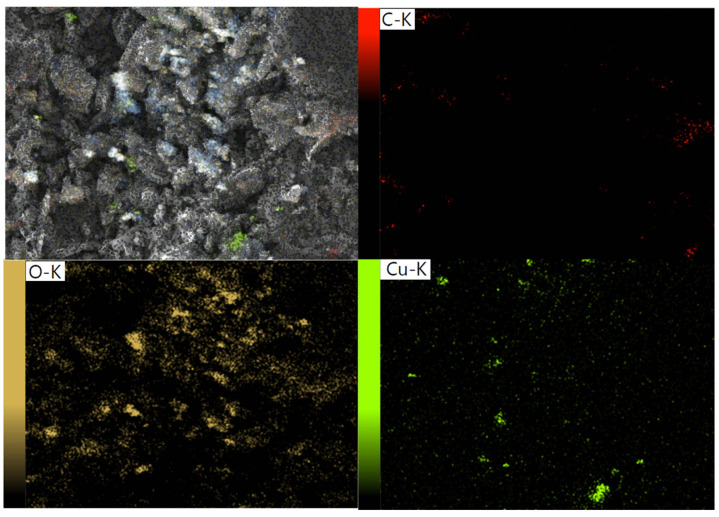
Mapping EDS of Cu/β-CD@CGO.

**Figure 4:**
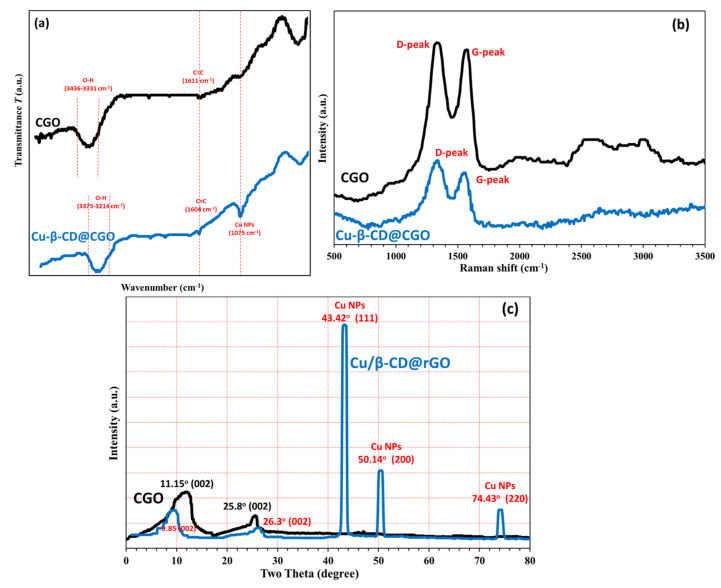
FTIR (**a**), Raman (**b**) and XRD (**c**) of Cu/β-CD@CGO.

**Figure 5:**
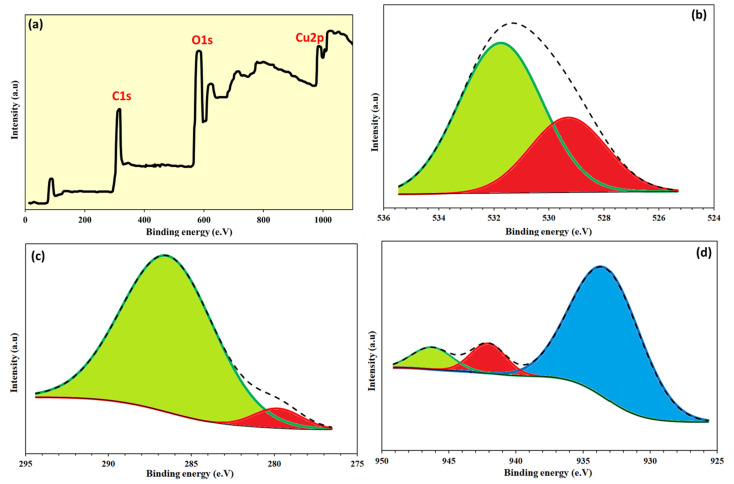
XPS spectra of Cu/β-CD@CGO; full scan (**a**), C1s (**b**), O1s (**c**), Cu2p (**d**).

The surface morphologies of CGO sheets and the Cu/β-CD@CGO were investigated using HRTEM and SEM; the resulting pictures are presented in Figure 2a–e. The SEM picture of CGO (Figure 2a) showed a vertical compact stack morphology and a characteristic structure that resembles a wrinkled sheet. In the Cu/β-CD/CGO SEM picture (Figure 2b), the β-CD moieties and supported Cu-NPs account for the tiny draping circles. Thin wrinkled films were seen in the 3D-CGO HRTEM picture (Figure 2c), which resulted from covalent interactions causing the CGO sheets to aggregate. The dark regions on the CGO sheets in the HRTEM picture of the Cu/β-CD/CGO (Figure 2d–f) exhibited β-cyclodextrin and Cu-NPs parts. The more haphazard 3D porous structure and thicker, rougher, and uneven surface of the 3D Cu/β-CD@CGO nanocomposite show that the doping of β-cyclodextrin and Cu NPs onto the CGO surface was successful. Using the DLS approach, the average diameter of the Cu/β-CD@CGO was found to be 37.5 ± 1.4 nm. Three distinctive peaks of Cu-nanoparticles were revealed by the EDX spectrograph of the Cu/β-CD@CGO nanocomposite (Figure S2). The first peak is located at 0.83–1.07 keV (Lα1), the next peak is of the highest intensity at 7.88–8.25 keV (Kα), and the last one is located at 8.68–9.11 keV (Kβ). To determine and map the C, O, and Cu elements in the Cu/β-CD@CGO and to assess the homogeneity and dispersion of Cu-dopants, mapping EDX was carried out (Figure 3). Figure 4a displays the CGO and Cu/β-CD@CGO FTIR. The Cu/β-CD@CGO and the CGO’s aromatic groups were found to have C=C vibrations, which were responsible for the absorption spectrum at 1604 cm^−1^ [32]. The β-CD functioning was demonstrated by 1031.5 cm^−1^ (R-1, 4-bond skeleton vibration of β-cyclodextrin) and the large absorption bands at 3214–3436 cm^−1^ (O-H stretching vibration) that were affixed to the nanocomposite’s surface. On the ternary nanocomposite, the supported Cu-nanoparticles exhibit a significant characteristic peak at 1075 cm^−1^. It is clear from FTIR spectroscopy that β-CD and Cu nanoparticles are immobilized on CGO nanostructures. Raman analysis was typically used to describe CGO faults and disorder states. Both CGO and Cu-β-CD@CGO displayed unique D (1391, 1385 cm^−1^) and G (1635, 1622 cm^−1^) bands in their Raman spectra (Figure 4b). The D band was attributed to vibrational stretching of the sp^3^ C-atoms associated with defects and disorder. The G band was generated by the E2g mode first-order scattering that resulted from the stretching vibration of the sp^2^ carbon atom [33,34]. Furthermore, the peak at 2755 cm^−1^ for CGO redshifted and broadened owing to the existence of the 2D overtone band [35], suggesting that some CGO sheet aggregation occurred. In contrast, the Cu-β-CD@CGO nanocomposite did not have this band, confirming the function of Cu-NPs in preventing CGO sheet aggregation. XRD was conducted to confirm the structural features of the Cu-β-CD@CGO (Figure 4c). The CGO exhibits a distinctive peak (002) at 11.15 degrees. Three distinctive diffraction peaks at 43.42° (111), 50.14° (200), and 74.43° (220) are present in the Cu-NPs in Cu-β-CD@CGO, and they are in excellent accord with the card for standard Cu [36,37,38,39]. The CGO sheet’s extra broad (002) peak at position 25 indicates a rise in crystallinity as compared to the earlier spectra [40,41,42,43]. The presence of carboxylic groups in the material may be the cause of this increase in crystallinity. The nanocomposite’s XRD spectrum shows no additional peaks, showing that the manufactured product is highly pure. Figure 5a–d illustrates the XPS analysis and the various interactions among CGO, β-cyclodextrin, and Cu-NPs moieties in the developed nanocomposite. Figure 5a shows the full scan spectrograph of Cu-β-CD@CGO, and the C, O, and Cu elements were distinctly identified. Figure 5b–d depict the C1s spectra of the Cu-β-CD@CGO, signifying a broad peak at 286.5 eV for the C-O, C=O, and O-C-O groups, as oxidized carbon species, which confirms the increased abundance of oxygen-containing functional groups in CGO. The diminutive peak at 279.8 eV indicated Cu-C bonding typical of carbide species. The O1s spectra of Cu-β-CD@CGO (Figure 5c) exhibit two prominent peaks at 531.8 and 529.2 eV, corresponding to C-O, O-C=O, C=O, and Cu-O bonds [44]. Three bands were visible in the Cu2p spectra in Figure 5d at 933.8, 942.2, and 946.4 eV. The presence of the Cu(II) oxidation state is confirmed by the other two minor bands, whereas the strongest broad band, located at 933.8 eV, is attributed to Cu-NPs (CuO) [45].

### 2.2. Analytical Parameter Optimization

#### 2.2.1. Desorption Solvent Impact

**Figure 6:**
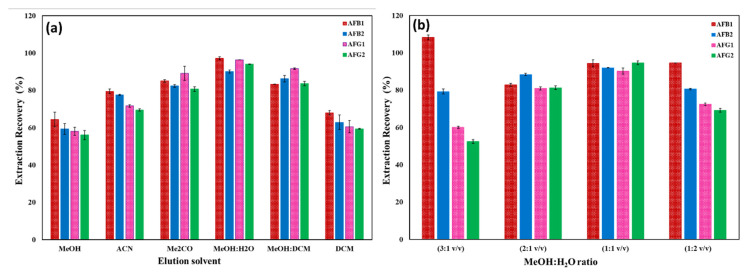
Impact of type of eluting solvent (**a**) and the volume ratio of MeOH/H_2_O (**b**).

To ensure a successful d-SPE analysis, the target analytes must be thoroughly cleaned off the nanosorbent surface. This outcome depends on the eluting solvent’s polarity and solubility of the analyte. MeOH, DCM, MeOH/DCM, ACN, Me_2_CO, and MeOH/H_2_O were tested to achieve a complete elution of AFs from the surface of Cu/β-CD@CGO, as displayed in Figure 6a. Cu/β-CD@CGO can interact with AFs through the hydrophilic part (O-atoms containing groups) and the hydrophobic part (benzene rings). These types of interactions included complex formation, H-bonds, dipole–dipole, and π-π stacking. Therefore, nonpolar (DCM) and a mixture of polar (ACN, Me_2_CO) eluting solvents were supposed to function better, but this is not the case. A mixture of equal volume ratio of methanol and water (1:1 *v*/*v*) was utilized to get the best elution of AFs. Cu/β-CD@CGO’s high electrostatic interactions with AFs are explained via complex-bonding attraction among the functional groups of oxygen in AFs and the Cu-NPs.

This indicates that these strong hydrophilic associations can be broken by eluting the AFs from the Cu/β-CD@CGO surface with a solvent of moderate to significant polarity. This demonstrates that complex formation is the main electrostatic interaction amongst Cu/β-CD@CGO and AFs. Thereafter, the proportion of eluting solvent between water and MeOH was further analyzed to determine optimal elution conditions (Figure 6b). Satisfactory AF recovery values were attained with methanol/water ratios of 1:2, 1:1, 2:1, and 3:1 (*v*/*v*). Ratios were applied to investigate the effect of elution volume on the extraction efficiency of aflatoxins. The results of this study revealed that 2.0 mL or more (2.0–5.0 mL) of MeOH/water (1:1 *v*/*v*) could attain recoveries of 93.8–100.6% for all the target AFs. The methanol/water (1:1 *v*/*v*) achieves optimal elution due to balanced polarity and disruption of Cu–O coordination. At 1.0 mL elution volume, the extraction recoveries and reproducibility were inadequate. Consequently, 2.0 mL of MeOH/water (1:1 *v*/*v*) solution was employed to elute AFs from the Cu/β-CD@CGO surface.

#### 2.2.2. pH Impact

**Figure 7:**
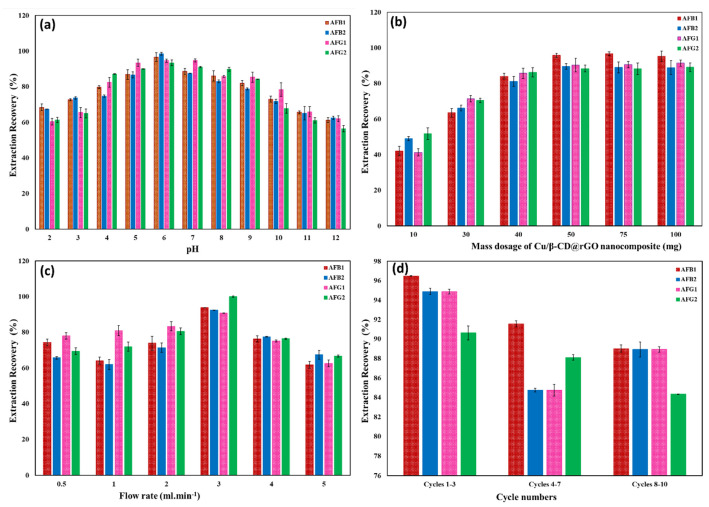
(**a**–**d**): Impact of pH (**a**); mass amount of nanocomposite (**b**); flow rate of AF solution (**c**); and Reusability analysis for AF extraction recoveries (**d**).

The AF solution pH directly affects the active sites of AFs and the stability of the nanosorbent, which in turn influences how well AFs can be removed. As the medium pH affects the protonation/deprotonation of functional groups for both AFs and the nanocomposite surface, the characteristics of the interaction are changed. Figure 7a depicts the pH influence on the AF removal efficiencies by Cu/β-CD@CGO in the range of 2–12. The AFs’ removal capacities were greater than 90% over the pH range of 5–7, and they attained their maximal value at pH 6. These experimental findings could be explained in terms of the development of a coordinate covalent bonding between the Cu-NPs and the lactone ring O-atoms in AFs, besides the inclusion complexes between the AFs and the β-CD moiety. Hydrophobic, π-π stacking interactions and H-bond formation with CGO sheets are considered the minor types of interactions. In highly basic (9–12) and acidic (1–3) pH values, the AFs’ lactone rings were opened, the type and structure of AFs were changed, and the AF removal recoveries were considerably reduced.

#### 2.2.3. Cu/β-CD@CGO Nanocomposite Mass Dosage Impact

During the d-SPE analysis, the extraction efficiency of the target analyte was directly affected by the mass of the nanosorbent. The 10–100 mg range of the Cu/β-CD@CGO was examined for AF removal efficiency at the optimum conditions. Figure 7b presents the results of this study. The maximum AF recovery was achieved with 50.0 mg of the developed nanocomposite, and no changes were observed when the nanocomposite mass was changed from 75.0 to 100 mg. This phenomenon can be explained by the high porosity with the huge CGO specific surface area and complex formation interactions with Cu-NPs. Furthermore, the Cu-NPs play a vital role in preventing the restacking of CGO nanosheets, thereby enhancing the available surface area for AF adsorption. The optimal quantity of Cu/β-CD@CGO to be packed into the extraction cartridge was determined to be 50.0 mg.

#### 2.2.4. Loading Flow Rate Impact

One important aspect that affects the extraction process is the flow rate for loading the sample, which also affects the time necessary for the analyte to adhere to the sites of binding on the sorbent. Figure 7c depicts the influence of aflatoxin flow rate on recovery results. Excellent recovery of AFs was achieved at 3.0 mL min^−1^ flow rates or below. Conversely, high flow rates resulted in low extraction recoveries because there was not enough time for AFs to fully interact with the Cu/β-CD@CGO nanocomposite’s binding sites. As a result, 3.0 mL. min^−1^ was selected as the flow rate for all investigations.

### 2.3. Cu/β-CD@CGO Reusability

The reusability of a nanosorbent is a critical criterion for assessing its overall performance. Under the optimized d-SPE conditions, the reusability of Cu/β-CD@CGO was examined through multiple successive extraction cycles. After each run, the nanocomposite was regenerated by flushing with 2.0 mL of MeOH:H_2_O (1:1, *v*/*v*), which provided efficient elution by balancing solvent polarity and disrupting Cu–O coordination sites. To further evaluate its robustness, the organic-solvent tolerance of Cu/β-CD@CGO was tested by immersing the material in methanol, acetonitrile, and dichloromethane for 48 h. No visible degradation or Cu leaching was observed, as verified by ICP-OES. Additionally, BET surface area and FTIR spectra remained unchanged, confirming excellent structural and solvent stability. Overall, the nanocomposite could be reused for up to 10 cycles with only a 4.3% variation in AF removal efficiency (Figure 7d). These results demonstrate that Cu/β-CD@CGO is a promising reusable sorbent and a potential alternative to immunoaffinity columns or conventional SPE cartridges.

### 2.4. Cu/β-CD@CGO Selectivity

**Figure 8:**
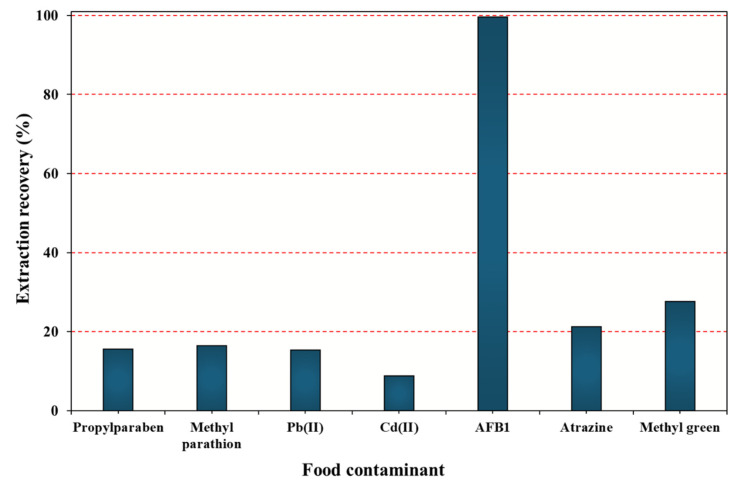
Selective response of Cu/β-CD@CGO nanocomposite to AFB1 and various prevalent dietary pollutants as interfering analytes under optimal conditions.

The selectivity of the Cu/β-CD@CGO nanocomposite was evaluated by comparing its extraction performance toward AFB_1_ with other common food contaminants representing different chemical classes, including preservatives (propylparaben), pesticides (methyl parathion and atrazine), heavy metals (Pb^2+^ and Cd^2+^), and a cationic dye (methyl green). As shown in Figure 8, all non-aflatoxin contaminants displayed markedly low extraction recoveries, generally below 20%, indicating weak or negligible affinity toward the nanocomposite. In contrast, AFB_1_ exhibited an extraction recovery close to 100%, demonstrating a significantly stronger interaction with Cu/β-CD@CGO than any of the competing analytes. This pronounced contrast highlights the inherent selectivity of the nanocomposite toward aflatoxins. The selective behavior can be attributed to multiple synergistic interactions: (i) host–guest inclusion of the planar hydrophobic aflatoxin structure within the β-cyclodextrin cavity, (ii) π–π stacking and hydrogen bonding interactions with the CGO surface, and (iii) coordination between electron-rich functional groups of AFB1 and the Cu^2+^ centers anchored onto the composite. Such combined mechanisms are not favored for structurally unrelated contaminants like parabens, organophosphate pesticides, metal ions, or cationic dyes, which explain their minimal uptake. Overall, the results confirm that Cu/β-CD@CGO possesses a strong and highly selective affinity toward aflatoxins, with negligible cross-reactivity toward other coexisting food contaminants. This selectivity makes the material particularly suitable for reliable extraction and enrichment of aflatoxins in complex food matrices.

### 2.5. Analytical Applications

The developed extraction method based on the Cu/β-CD@CGO nanocomposite was used to evaluate the linearity, recovery, accuracy, LOD, and LOQ of the AF extraction. An ANOVA tests were accompanied by a *p*-value < 0.05, showing no significant difference between the AFs’ standard curves produced by solutions with sample matrix-matching and those formed from standard solutions. With all certified samples having an interval of confidence of 95%, the *t*-test (*p*-values > 0.05) and z-test (z-values 1.95) results demonstrated that the approach was sufficiently accurate to detect the specifically targeted aflatoxins in maize, pistachio, and rice samples. There was no discernible difference between the AF readings in the recommended method and the approved materials. This suggests that external calibration can be employed, as the Cu/β-CD@CGO nanocomposite may remove the interfering and matrix substances throughout the analysis. Maximum allowable limits for AFs in maize, pistachio and rice (10.0 µg kg^−1^ for total AF content, 5.0 µg kg^−1^ for AFB1 and 8.0 µg kg^−1^ for AFB1 in pistachio) are specified by the European Community Regulation (EC), which were satisfied by the limits of quantification (LOQs) for four AFs, extending from 0.0185 to 0.0232 µg kg^−1^. For all target AFs, the relative standard deviations within and between days were smaller than 4.1% and 5.3%, respectively. Recovery tests were conducted to assess accuracy (Table 1). For triplicate runs, the RSDs extended from 0.43% to 4.2%, while the recovery percentages ranged from 92.0% to 108.5%. Consequently, the suggested technology can be used to accurately and formally control trace levels of AFs in various foods. In Figure S1, the HPLC-FLD chromatogram AFG1, AFG2, AFB2, and AFB1 aflatoxins (10 ng mL^−1^ at flow rate of 1.0 mL min^−1^) in 2.0 mL of methanol: water (1:1 *v*/*v*) are shown.

### 2.6. AF Adsorption Mechanism

**Figure 9:**
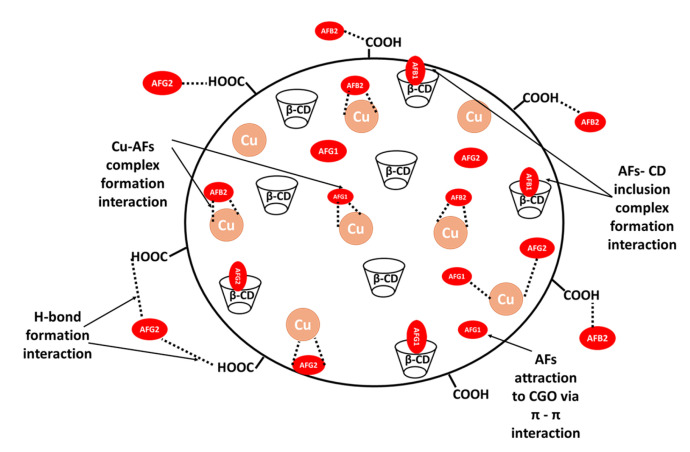
Different possible binding interactions responsible for the AF adsorption onto the surface of Cu/β-CD@CGO nanocomposite.

Generally, the porosity of Cu/β-CD@CGO improves AF interaction with its binding active sites by enabling efficient mass transfer at elevated rates. AF adsorption onto the surface of Cu/β-CD@CGO is controlled by various AFs-Cu/β-CD@CGO interactions, including hydrogen bonding, π–π interactions, metal-complex formation, and inclusion complex formation interactions (Figure 9). Four different mechanistic forms were anticipated for the extraction of AFs with Cu/β-CD@CGO, dependent on the medium pH. The first one is the complex formation process between doped copper nanoparticles and the lactone ring O-atoms in AFs, and this is considered the main electrostatic attraction for AF uptake by Cu/β-CD@CGO. The second mechanism is governed by the trapping of the target AFs into the β-CD moiety’s hydrophobic central cavity to give inclusion complexes. The interactions concerning the π-electrons of CGO sheets and the π-cloud of AF aromatic rings, which are referred to as π-π interactions, are the third mechanism [46,47,48,49]. This factor directs the adsorption and deposition of porphyrin and the nucleobases of the ssDNA ring structure onto graphene sheets [50,51,52,53,54,55,56,57,58]. The fourth mechanism is the electrostatic interactions and H-bonding that are formed between the huge number of carboxyl groups in CGO and the oxygen functional groups in AFs. Nevertheless, the development of Cu-AF complexes as well as inclusion complexes with β-CD plays a crucial role in improving the Cu/β-CD@CGO nanocomposite’s adsorptive absorption of AFs.

### 2.7. Comparison of Other Reported SPE-Based Techniques with the Cu/β-CD@CGO Approach

Table 2 presents a comparison of the proposed strategy with conventional d-SPE-based methodologies for determining the amount of aflatoxins in various food products. In terms of % recovery (accuracy), %RSD (precision), and LODs (sensitivity), the analytical performance of the method based on Cu/β-CD@CGO was excellent. Besides, the developed method provided for AF recoveries was better in the LODs and recovery values for other approaches. This advantage is explained by Cu-NPs’ exceptional ability for complex formation with AFs and the porous CGO’s enormous specific surface area. In addition, the reusability, stability, and simplicity of using the nanocomposite make the developed approach have certain advantages over IAC methods. Based on these experimental findings, the Cu/β-CD@CGO nanocomposite exhibits considerable promise for recovering AFs from complicated matrices.

## 3. Conclusions

This work introduces a newly designed Cu/β-CD@CGO nanocomposite that establishes a distinctive, multidimensional adsorption framework for the microextraction of four major aflatoxins from complex cereal and nut matrices. Synthesized through a simple and green route, the hybrid sorbent combines three complementary interaction domains—Cu–lactone coordination, β-cyclodextrin host–guest inclusion, and carboxylated graphene oxide π-rich and hydrogen-bonding interfaces—to generate a synergistic platform not achievable with conventional graphene- or β-CD–based sorbents. This structural integration markedly improved selectivity, sorption capacity, and resistance to nanosheet restacking, setting the material apart from previously reported Cu/β-CD/graphene systems. Under optimized d-SPE conditions, the Cu/β-CD@CGO sorbent provided excellent analytical performance, including high recoveries (92.0–108.5%), broad linearity (0–20 µg kg^−1^), and ultralow detection limits down to 0.0062 µg kg^−1^ for all target aflatoxins. The nanocomposite-maintained extraction efficiency over 10 reuse cycles and required only minimal solvent volumes, demonstrating its operational robustness and sustainability. Its ability to effectively remove matrix interferences in pistachio, maize, and rice further underscores its suitability for real-world applications. Collectively, these findings position Cu/β-CD@CGO as a next-generation graphene-based sorbent that advances aflatoxin monitoring by uniting high sensitivity, chemical selectivity, environmental compatibility, and reusability. The platform provides a promising alternative to immunoaffinity cleanup and represents a valuable analytical tool for high-throughput, routine food safety surveillance.

## 4. Experimental Section

### 4.1. Materials and Reagents

A Sinopharm Chemical Reagent Co., Ltd. (Shanghai, China) delivered the graphene oxide powder. HPLC-grade solvents were supplied from Merck Chemicals (Darmstadt, Germany), including methanol (MeOH), acetone (Me_2_CO), acetonitrile (ACN), sodium chloride (NaCl), and anhydrous copper (II) chloride (CuCl_2_). R-Biopharm AG delivered the acquired immunoaffinity (IA). Typical solutions of AFG2, AFG1, AFB2, AFB1 at a concentration of 10.0 mg L^−1^ were prepared in an equal volume ratio of MeOH/water mixture and refrigerated at 4 °C. Daily measured solutions were made from stock one before use by diluting the stock solution with 18.0 M cm resistivity water to the appropriate concentrations.

### 4.2. Instruments

#### 4.2.1. HPLC-FLD

Aflatoxins were detected using an Agilent 1260 Infinity II HPLC system equipped with a post-column photochemical reactor (PHRED, UVE™) to promote fluorescence enhancement prior to FLD detection at excitation/emission wavelengths of 362/455 nm. Before utilization, the mobile phase comprising 70% H_2_O, 15% methanol, and 15% acetonitrile was filtered via a micro-filtration medium.

#### 4.2.2. Characterization Techniques

A JEOL scanning electron microscope (SEM- JSM-6010LV) is used to check the morphology of the GO, CGO, and Cu/β-CD@CGO nanoparticles. At different magnification levels, the morphology of the Cu/β-CD@CGO nanocomposite was examined using a transmission electron microscope with high resolution (HRTEM, JEOL-JEM 2100, Japan). The patterns of X-ray diffraction (XRD) were obtained between 10° and 70° in angular range using a D8 ADVANCE diffractometer. The valence state and chemical composition of Cu/β-CD@CGO were analyzed using X-ray photoelectron spectroscopy (XPS, Thermo ESCALAB 250Xi, East Grinstead, UK). Cation concentrations were measured using a D/MAX-2550-Rigaku-X-ray powder diffractometer, with a source of Cu-Kα radiation to characterize the fabricated nanocomposite. Fourier transform infrared (FTIR-Nicolet 400, Germany) spectrophotometer was used to detect the functional groups between 4000 and 400 cm^−1^. To calculate the BET pore size and surface area, as well as to monitor the N_2_ adsorption/desorption isotherm, a Physisorption Nova3200-N equipment was utilized.

### 4.3. Chromatographic Conditions

The autosampler unit was used to inject 0.75 µL of sample at a 1.0 mL min^−1^ flow rate into the analytical column. The column’s temperature was 60 °C. 0.45 mL min^−1^ was the post-column derivatization reagent’s flow rate. In chromatographic conditions, a pure mixture of the four AFs (20 to 0.25 ng L^−1^) was introduced to produce the chromatogram of HPLC-FLD, illustrating the corresponding retention times of the resolved peaks [69,70]. Figure S1 displays the HPLC-FLD chromatogram of AFG1, AFG2, AFB2, and AFB1 in 2.0 mL of methanol/water (1:1 *v*/*v*). The four AFs that were found have their retention duration (min), emission fluorescence wavelength (nm), and UV excitation wavelength (nm) listed in Table S1.

### 4.4. Cu/β-CD@CGO Nanocomposite Synthesis

The Cu/β-CD@GO nanocomposite was prepared via a green approach through hydrothermal generation of copper nanoparticles and then doping into the β-CD-functionalized CGO using a ball mill reactor. Firstly, the oxygen functional groups (epoxy and hydroxyl groups) at the basal and edged planes of GO were carboxylated to give CGO by adding 30.0 g of NaOH and 25.0 g of chloroacetic acid to 5.0 g of GO [71]. The suspended mixture was then ultrasonically agitated for five hours. The CGO black precipitate was filtered, washed with methanol and water, and then vacuum-dried at 70 °C. Secondly, Cu-NPs were synthesized hydrothermally in a stainless-steel autoclave using CuCl_2_ as the precursor and L-ascorbic acid as the reducing agent [72]. The pH was modified to 11 utilizing 0.1 M NaOH. In a ball mill, the synthesized Cu-NPs (0.20 g) were finally mixed with 0.20 g β-CD and 0.50 g CGO. The solid mixture was then ground at a frequency of 25 Hz for 30 min.

### 4.5. Treatment of Real Samples

**Figure 10:**
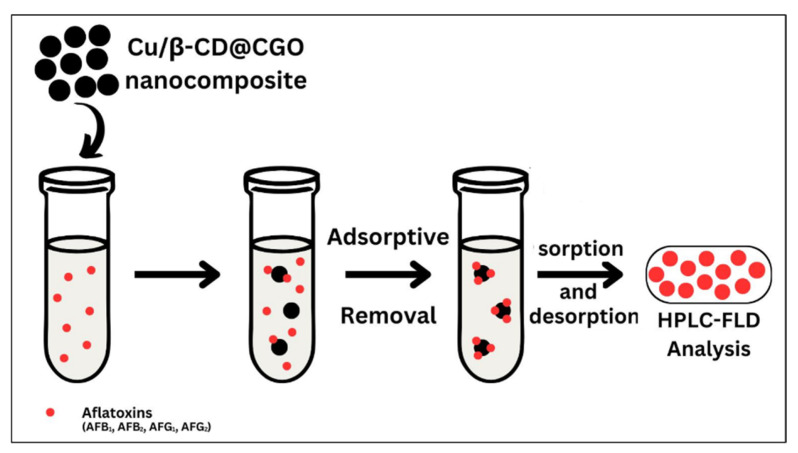
Schematic illustration of the d-SPE process used to extract AFs using the Cu/β-CD@CGO.

Samples of rice, pistachios, and maize were purchased from local markets in Saudi Arabia and homogenized into a fine powder using a standard blender. For extraction, approximately 5.0 g of each powdered sample was mixed with 40 mL of MeOH/H_2_O (80:20, *v*/*v*), ultrasonicated for 15 min, and centrifuged. The resulting filtrate (15 mL) was passed through the Cu/β-CD@CGO SPE cartridge at a flow rate of 3.0 mL min^−1^. 5.0 g of sample was combined with 40 mL of MeOH/H_2_O (4:1, *v*/*v*) and 5.0 g NaCl in a 50 mL tube, followed by 15 min ultrasonic extraction [26]. After centrifugation for 5 min, the supernatant was filtered, and its initial pH (≈7.2) was adjusted to pH 6.0 using 0.1 M HCl. A 15 mL aliquot of this extract was also loaded onto the Cu/β-CD@CGO cartridge at 3.0 mL min^−1^. The sorbent was rinsed with 10 mL of MeOH/ACN to remove co-adsorbed matrix components. Elution of the retained aflatoxins was achieved using 2.0 mL of MeOH/H_2_O (1:1, v/v), and the eluate was immediately analyzed by HPLC-FLD for chromatographic identification and quantification. A schematic overview of the SPE workflow using the Cu/β-CD@CGO nanocomposite is provided in Figure 10.

## Figures and Tables

**Table 1:** Analysis results of AFs in pistachio, maize, and rice samples using the proposed Cu/β-CD@CGO-based microextraction approach.

Analyte	Rice				Maize					Pistachio		
	Added (μg kg^−1^)	Found (μg kg^−1^)	%*R* ^a^	RSD ^b^ (*n* = 3)	Added (μg kg^−1^)	Found (μg kg^−1^)	%*R* ^a^	RSD ^b^ (*n* = 3)	Added (μg kg^−1^)	Found (μg kg^−1^)	%*R* ^a^	RSD ^b^ (*n* = 3)
AFG1	0	ND ^c^			0	ND			0	ND	-	-
	0.25	0.23	93.5	2.13	0.25	0.23	96.8	2.06	0.25	0.24	97.5	2.05
	0.50	0.46	92.0	0.43	0.5	0.48	94.4	0.42	0.50	0.48	96.0	0.41
	2.0	1.90	95.0	1.57	2.0	1.92	96.1	1.56	2.00	1.92	96.0	1.56
	20.0	21.11	106	1.40	20.0	21.3	107	2.05	20.0	21.4	107	3.33
AFG2	0	ND			0	ND			0	ND		
	0.25	0.23	92.5	2.16	0.25	0.25	95.5	2.09	0.25	0.24	96.5	2.00
	0.50	0.46	92.8	1.21	0.5	0.47	101	3.96	0.50	0.48	108	4.31
	2.0	1.89	94.8	2.63	2.0	1.91	95.8	2.61	2.00	1.92	96.0	2.60
	20.0	19.9	99.5	2.01	20.0	20.3	102	1.97	20.0	21.4	103	1.93
AFB1	0	ND			0	ND			0	ND		
	0.25	0.24	104	4.08	0.25	0.25	104	4.08	0.25	0.25	108	4.08
	0.50	0.51	96.0	4.16	0.5	0.52	102	3.92	0.50	0.54	100	4.00
	2.0	2.05	102	0.97	2.0	2.08	104	0.96	2.00	2.07	104	0.96
	20.0	20.89	104	0.74	20.0	21.1	106	1.07	20.0	21.4	107	3.41
AFB2	0	ND			0	ND			0	ND		
	0.25	0.24	105	1.89	0.25	0.23	103	1.93	0.25	0.24	107	1.87
	0.50	0.52	97.0	4.12	0.50	0.51	95.0	4.21	0.50	0.53	99.0	4.04
	2.0	2.06	103	2.42	2.00	2.06	103	2.42	2.00	2.08	104	2.40
	20.0	21.24	106	1.41	20.0	21.0	105	1.42	20.0	21.5	108	1.39

^a^ Recovery %. ^b^ Relative standard deviation. ^c^ ND: Not detected.

**Table 2:** Analytical performance comparison of the proposed approach with previously documented SPE methods for the analysis of aflatoxins in various cereal meals.

Method	Sample Matrix	Adsorbent Type	LOD (µg kg^−1^)	Recovery (%)	RSD (%)	Adsorbent Amount (mg)	Reference
SPE/HPLC–DAD–FLD	Wheat, Barley, Rye, Maize, Oat, Rice	Oasis HLB cartridges	0.03–0.09	90.1–112	<7.20 & <11.9	–	[46]
MSPE ^a^/HPLC-FLD	Corn, Rice	AMT/TMSPT MNPs ^b^	B1:0.014, 0.150 B2: 0.05, 0.05	90.3–97.0	<4.65 & <4.97	150	[47]
SPE/HPLC-FLD	Wheat, Rice, Oat, Barley	hyperbranched polymer	0.01–0.12	82.7–103	<10	50	[59]
SPE/HPLC-FLD	Rice, Maize	LiChrolut C18 cartridge	0.02–0.03	89.2–97.8	<1.64 & <2.79	200	[60]
SPE/HPLC-FLD	Corn, Wheat Oasis	HLB and Bond Elution cartridges	Corn: 0.05–0.08 Wheat: 0.04–0.07	90.7–106	<6.40 & <15.8	–	[61]
IAC/HPLC-FLD	Rice, Wheat, Oat, Barley, Corn	AOZ-IAC	0.004–0.012	77.3–104	<14.3 & <15.2	–	[62]
MSPE/LC-MS	Corn, Wheat	mGCB ^c^	0.05–0.10	63–78	<12.0 & <20.0	–	[63]
SPE-DLLME/HPLC-FLD	Cereal, beans and oil	C-18	0.03–11.0	63.2–108	<8.13	500	[64]
DSPE ^d^/HPLC-FLD	Pistachio, Rice	Fe_3_O_4_	0.06–0.35	76.0–112.7	<150	50	[65]
SPE/HPLC-FLD	Maize, Rice	Silica/GO	0.10–0.30	76.8–106.9	<3.90 & <6.40	100	[66]
SPE/HPLC-FLD	Soybean and soy-based food	3D-graphene @ Fe_3_O_4_	0.09–0.15	83–103	<3.40 & <7.50	30	[67]
SPE/HPLC-FLD	Maize, Cereal-based feed	β-CD porous graphene	0.01–0.03	90.5–105	<3.70 & <6.10	15	[68]
d-SPE/HPLC-FLD	Rice, Pistachio and Maize	Cu@ graphene nanocomposite	0.018–0.02 (AFB1, AFB2, AFG1, AFG2)	92.3–109%	0.43–4.20	50	This work

^a^ Magnetic solid phase extraction. ^b^ 2-amino-5-mercapto-1,3,4-thiadiazole/3-(trimethoxysilyl)-1-propanthiol magnetic nanoparticles. ^c^ Magnetic graphitized carbon black. ^d^ Dispersive solid phase extraction.

## Data Availability

The original contributions presented in this study are included in the article/Supplementary Material. Further inquiries can be directed to the corresponding author(s).

## Supplementary Materials

The following supporting information can be downloaded at: https://www.mdpi.com/article/10.3390/toxins17110562/s1, Figure S1: HPLC-FLD chromatogram of AFG1, AFG2, AFB2, and AFB1 aflatoxins (10 ng mL^−1^ at flow rate of 1.0 mL min^−1^) in 2.0 mL of methanol: water (1:1 *v*/*v*); Figure S2: EDX of Cu/β-CD@CGO nanocomposite; Table S1: Identification of aflatoxins in HPLC-FLD.
