# Supplementary material for: Trace Aflatoxins Extraction in Pistachio, Maize and Rice Based on β-Cyclodextrin-Doped Cu-Carboxylated Graphene Oxide Nanocomposite
Source: Toxins (Basel). 2025 Nov 17;17(11):562. doi: 10.3390/toxins17110562 (PMC12656133; doi:10.3390/toxins17110562)
Supplement: Supplementary file 1 [file toxins-17-00562-s001.zip › toxins-3948032-supplementary.pdf]

toxins

MDPI

Article

Trace Aflatoxins Extraction in Pistachio, Maize and Rice Based on β-Cyclodextrin-Doped Cu-Carboxylated Graphene Oxide Nanocomposite

Amr A. Yakout [1,2,*], Wael H. Alshitari [1], Hassan M. Albishri [3], Faten M. Ali Zainy [1] and Adel M. Alshutairi [3,*]

1 Department of Chemistry, College of Science, University of Jeddah, Jeddah 23218, Saudi Arabia
2 Chemistry Department, Faculty of Science, Alexandria University, Alexandria 21526, Egypt
3 Department of Chemistry, Faculty of Science, King Abdulaziz University, P.O. Box 80203, Jeddah 21589, Saudi Arabia
* Correspondence: aayhassain@uj.edu.sa (A.A.Y.); aalshutairi0001@stu.kau.edu.sa (A.M.A.)

Supplementary Materials

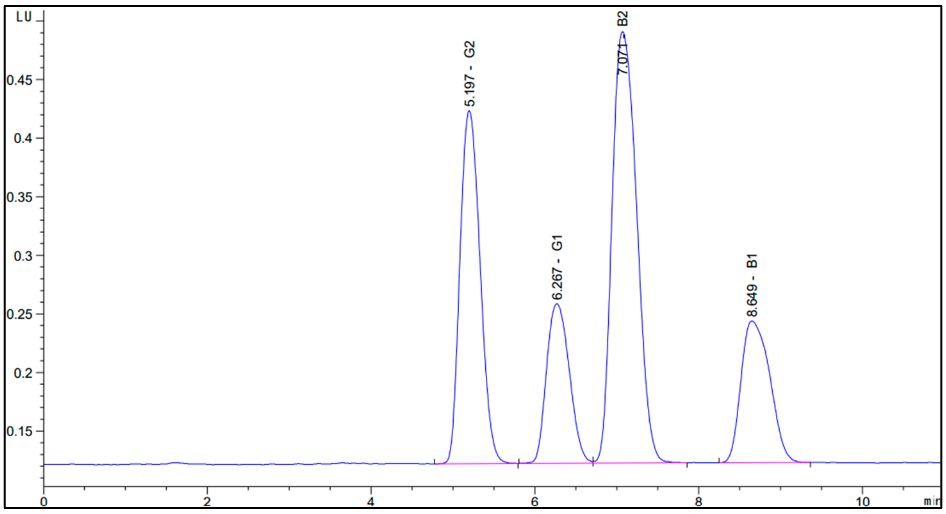

Figure S1. HPLC-FLD chromatogram of AFG1, AFG2, AFB2, and AFB1 aflatoxins (10 ng mL-1 at flow rate of 1.0 ml min-1) in 2.0 mL of methanol: water (1:1 v/v).

Received: 9 October 2025
Revised: 9 November 2025
Accepted: 13 November 2025
Published: 17 November 2025

Citation: Yakout, A.A.; Alshitari, W.H.; Albishri, H.M.; Zainy, F.M.A.; Alshutairi, A.M. Trace Aflatoxins Extraction in Pistachio, Maize and Rice Based on β-Cyclodextrin-Doped Cu-Carboxylated Graphene Oxide Nanocomposite. *Toxins* **2025**, *17*, 562. https://doi.org/10.3390/toxins17110562

Copyright: © 2025 by the author. Licensee MDPI, Basel, Switzerland. This article is an open access article distributed under the terms and conditions of the Creative Commons Attribution (CC BY) license (https://creativecommons.org/licenses/by/4.0/).

Table S1. Identification of aflatoxins in HPLC-FLD.

Analyte	UV absorption (nm)	Fluorescence Emission (nm)	Retention time (min)
AFG1	243	450	6.26 (± 0.7)
AFG2	265	450	5.17 (± 0.7)
AFB1	223	425	7.07 (± 0.7)
AFB2	265	425	8.64 (± 0.7)

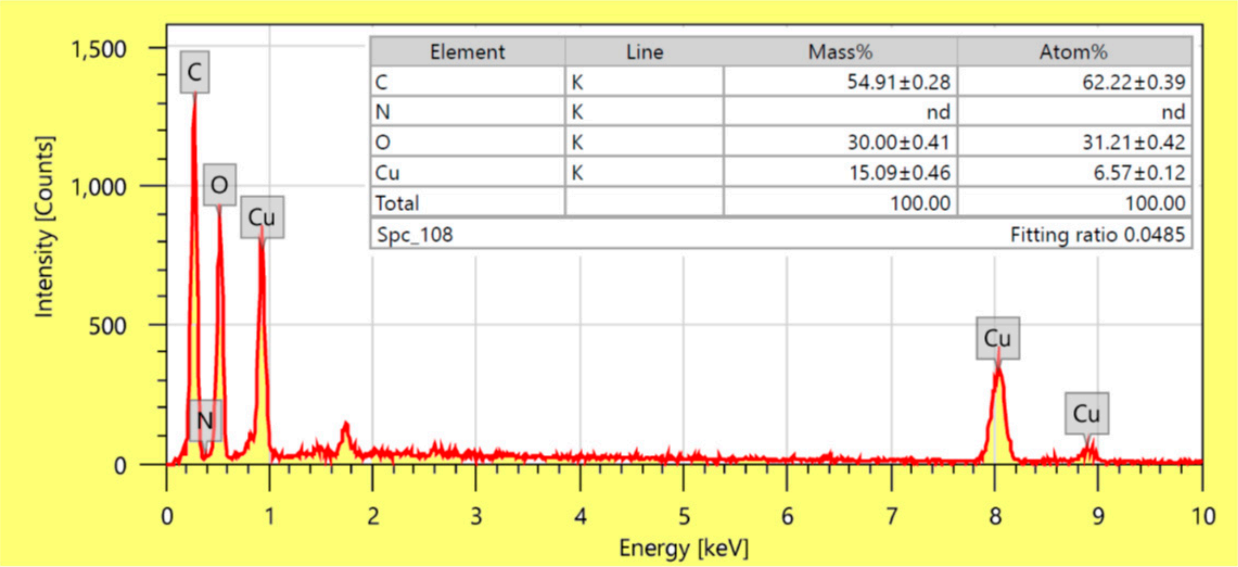

Figure S2. EDX of Cu/β-CD@CGO nanocomposite.
